# Material deprivation and unemployment affect coercive sex among young people in the urban slums of Blantyre, Malawi: A multi-level approach

**DOI:** 10.1016/j.healthplace.2015.03.001

**Published:** 2015-05

**Authors:** Mphatso Kamndaya, Lawrence N. Kazembe, Jo Vearey, Caroline W. Kabiru, Liz Thomas

**Affiliations:** a School of Public Health, University of the Witwatersrand, 27 St Andrews Road, Parktown, 2193 Johannesburg, South Africa; b Department of Statistics & Population Studies, University of Namibia, Windhoek, Namibia; c African Centre for Migration and Society, School of Social Sciences, University of the Witwatersrand, Johannesburg, South Africa; d Population Dynamics and Reproductive Health Research Program, African Population and Health Research Center, Nairobi, Kenya

**Keywords:** Urban slums, Coercive sex, Young people, Material deprivation, Unemployment

## Abstract

We explore relations among material deprivation (measured by insufficient housing, food insecurity and poor healthcare access), socio-economic status (employment, income and education) and coercive sex. A binary logistic multi-level model is used in the estimation of data from a survey of 1071 young people aged 18–23 years, undertaken between June and July 2013, in the urban slums of Blantyre, Malawi. For young men, unemployment was associated with coercive sex (odds ratio [OR]=1.77, 95% confidence interval [CI]: 1.09–3.21) while material deprivation (OR=1.34, 95% CI: 0.75–2.39) was not. Young women in materially deprived households were more likely to report coercive sex (OR=1.37, 95% CI: 1.07–2.22) than in non-materially deprived households. Analysis of local indicators of deprivation is critical to inform the development of effective strategies to reduce coercive sex in urban slums in Malawi.

## Highlights

• Local measures were used for an analysis of association between deprivation and coercive sex.
• Unemployment was associated with coercive sex among young men.
• Material deprivation was associated with coercive sex among young women.

## 1 Introduction

Coercive sex – being forced by someone (partner or non-partner) to have sex when one does not want to – is increasingly visible on the public health agenda worldwide. Coercive sex represents a gross violation of young people׳s sexual rights and increases their risk for sexually transmitted infections, including HIV infection, and unplanned pregnancy (World Health Organization, 2013). A growing body of research in sub-Saharan Africa has highlighted the high risk for both partner and non-partner coercive sex among young people residing in growing urban poor communities – urban slums (Decker et al., 2014; Misganaw and Worku, 2013; Oduro et al., 2012). Therefore, reliable research on contextual factors that increase their vulnerability to coercive sex is required in order to inform effective strategies targeting this increasingly marginalized segment of young people. We explore relations among material deprivation, socio-economic status (SES) variables and coercive sex among young men and young women in two urban slums of Blantyre, Malawi.

Malawi is a south-east African country of about 16 million people (Population Reference Bureau [PRB], 2013), with a gross national income per capita of $350 (World Bank, 2010). It is estimated that around 70% of Malawi׳s urban population lives in urban slums – areas defined as lacking more than one of the following: access to safe water, adequate sanitation, sufficient living space, durability of housing, and security of tenure (UN-Habitat, 2011). Eleven percent of the Malawian population is living with HIV (urban 17.4%, rural 8.9%); HIV prevalence among young women aged 15–24 years is 2.7 times that among young men, at 5.2% and 1.9% respectively (National Statistical Office of Malawi and ICF Macro, 2011).

**Fig. 1:**
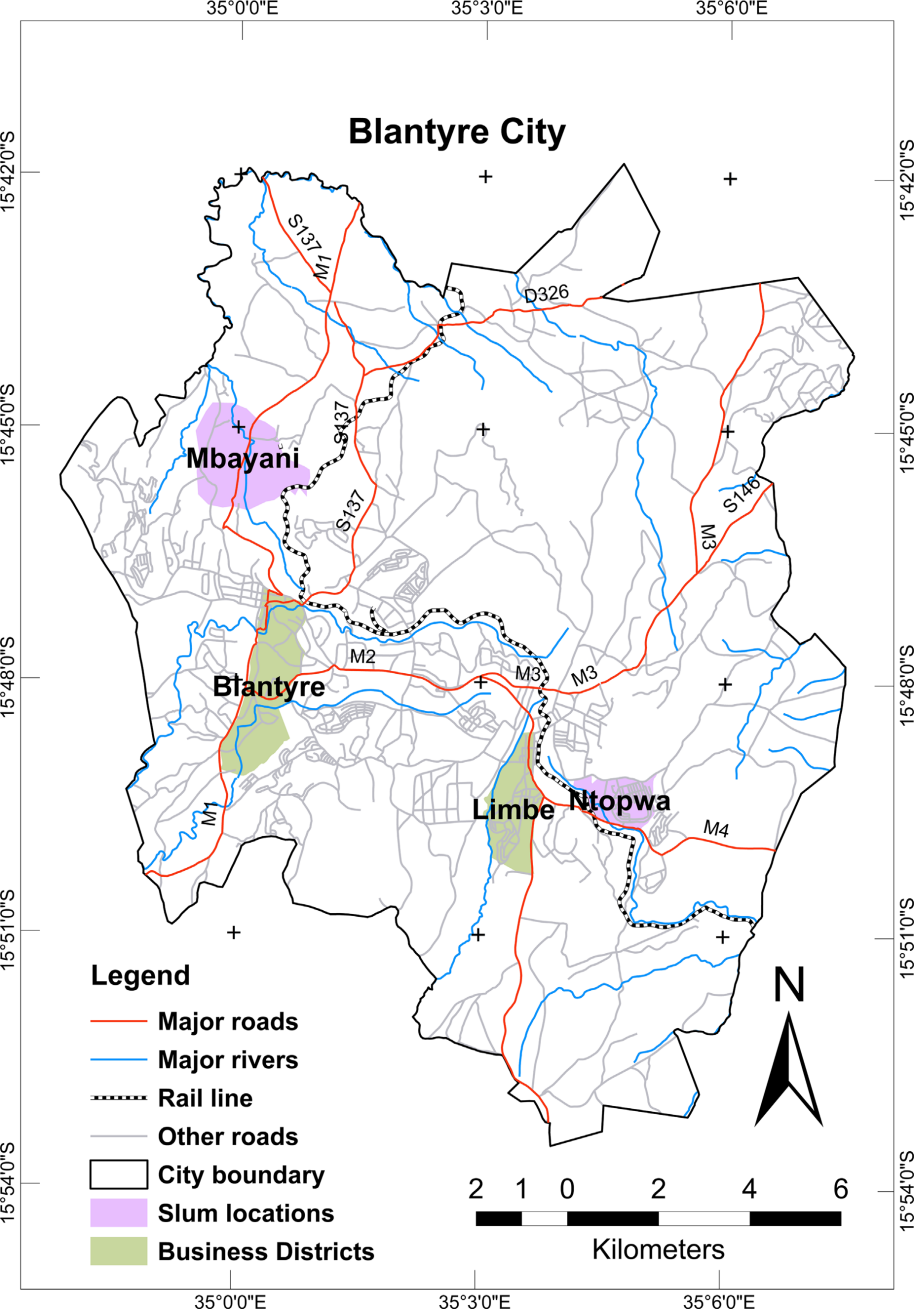
Map of Blantyre City showing the two study locations.

Blantyre is a helpful example of a currently urbanizing space, typical of other areas within sub-Saharan Africa where urban spaces are growing. The urban population in Blantyre is estimated to be 816,001, by 2013, which is an eightfold increase since 1966, with the then population at 106,641(National Statistical Office of Malawi, 2008). An estimated 60% of the urban population in Blantyre reside in urban slums where many young people may have no education or job skills (Mughogho and Kosamu, 2012; UN-Habitat, 2011). Given their limited resources and lack of opportunities, some young people engage in desperate survival strategies – such as exchanging sex for basic necessities including access to housing, food and healthcare (Kamndaya et al., 2015; Mandalazi et al., 2013). Blantyre City started with developments in three commercial centers – Blantyre, Mandala and Limbe – in 1876 by the Church of Scotland. With time, Mandala became integrated into Blantyre commercial centre, bringing the number of commercial centers within the city to two – Blantyre and Limbe (Mughogho and Kosamu, 2012). Against this backdrop, we selected two urban slums close to these two commercial centers (Mtopwa close to Limbe commercial centre, and Mbayani close to Blantyre commercial centre) (see, Fig. 1) in order to explore differences between the experiences of young people residing in these different locations, highlighting examples of intra-urban inequalities and the role of place on coercive sex within the city. The selection of the two urban slums is also based on the literature that suggests that Mbayani and Mtopwa are broadly typical of urban slums not only in Blantyre, but Malawi (Chipeta, 2009).

Studies undertaken in Malawi have found clustering of coercive sex within households and neighborhoods (Kathewera-Banda et al., 2005; Mason and Kennedy, 2014), but limited attention has been paid to exploring variations across neighborhoods in urban slums. Factors associated with coercive sex in Malawi include individual socioeconomic status, marital status and urban/rural residence (Conroy and Chilungo, 2014; Kathewera-Banda et al., 2005; Mason and Kennedy, 2014; Moore et al., 2012; National Statistical Office of Malawi and ICF Macro, 2011). However, neighborhood socioeconomic factors have an overarching influence on sexual violence (Fox and Benson, 2006; Hindin and Adair, 2002; Jewkes, 2002; Kiss et al., 2012; McIlwaine, 2013). For instance, limited access to resources or lack of opportunities may prompt young people to find a way to support themselves (Cunradi et al., 2000; Fox and Benson, 2006; Pearlman et al., 2003). In order to survive, some young people may resort to transactional sexual relationships that increase their vulnerability to coercive sex (Mulumeoderhwa and Harris, 2014; Sikweyiya and Jewkes, 2009; Tyler and Johnson, 2006). The social aspects of the neighborhood – for example, weak social cohesion and collective efficacy – can also impart risk of coercive sex (Frye et al., 2014; Pinchevsky and Wright, 2012). Although the possible neighborhood characteristics associated with coercive sex are considered to be multiple and diverse, measures of material deprivation have attracted a lot of research interest particularly in the growing urban poor communities in low-income countries (Chant, 2013; McIlwaine, 2013). Decker et al. (2014) and Pandey et al. (2009) have provided a helpful explanation for this interest – that residents in urban poor communities are more likely to become victims of coercive sex because of the wide range of social vulnerabilities associated with living in resource-poor settings including weak social ties, limited police protection, pervasive violence, and limited access to social and health resources. Gaining a clearer understanding of these potential relations not only contributes to the growing knowledge around the role of material conditions in coercive sex but also can be a useful guide to policy making in growing urban poor communities in sub-Saharan Africa.

## 2 Background

A growing body of research worldwide has documented substantial numbers of young men and young women who have experienced coercive sex in their lifetimes (Andersson et al., 2012; Erulkar, 2004; Hines, 2007; Kuyper et al., 2013; Meinck et al., 2015; Sikweyiya and Jewkes, 2009). For example, research conducted by Hines (2007) across 38 research sites in high income countries found that overall 2.3% of young women and 2.8% of young men reported having experienced coercive sex. Similarly, Andersson et al. (2012) found that overall 19.6% of young women and 21.1% of young men reported experience with coercive sex in a study across 10 countries (including Malawi) in sub-Saharan Africa. Although coercive sex is experienced by both genders, gender differences exist in young women׳s experiences of coercive sex and those of young men (Sikweyiya and Jewkes, 2009), supporting the idea of considering gender differences in the analysis of risk factors and prevention strategies.

Individual risk factors of coercive sex are well-established. Notably, young age at first sex (Andersson et al., 2012; Bazargan-Hejazi et al., 2013; Koenig et al., 2004; Moore et al., 2007), substance use (Conroy and Chilungo, 2014; Kabiru et al., 2010; Koenig et al., 2004; McCrann et al., 2006; Zablotska et al., 2009), and having multiple sexual partners (Bingenheimer and Reed, 2014; Moore et al., 2007; Moore et al., 2012) are associated with coercive sex. Individual SES and marriage have also been associated with coercive sex (Adudans et al., 2011; Conroy and Chilungo, 2014; Koenig et al., 2004; Ybarra et al., 2012; Zablotska et al., 2009), although the results are found to be contradictory. For example, Adudans et al. (2011), found that young women with high economic status or post-primary education in Kenya were more likely to report having experienced coercive sex, while Koenig et al. (2004) found no association between individual SES factors and coercive sex in rural Uganda. In view of these equivocal findings, the interest in research around the world on associations between socioeconomic indicators and coercive sex has grown.

### 2.1 Disadvantage, urban slums, and coercive sex

Structural characteristics of poor neighborhoods including disadvantage can heighten risk of sexual violence in some settings (Cunradi et al., 2000; Fox and Benson, 2006; Kiss et al., 2012; Pandey et al., 2009; Pearlman et al., 2003; Pinchevsky and Wright, 2012) in a general way that could be applied to coercive sex as well. We focus here on measures of disadvantage that have been found to be most critical in urban slums in sub-Saharan Africa – insufficient housing, food insecurity and poor access to healthcare (Kamndaya et al., 2014; Greif, 2012; Thomas et al., 2011; Vearey et al., 2010). McIlwaine (2013) notes that insufficient housing, food insecurity and poor access to healthcare, and other indicators of disadvantage intensify or attenuate the effects of individual-level factors on coercive sex particularly in urban slums. However, rigorous research is needed in urban slums in sub-Saharan Africa to substantiate McIlwaine׳s (2013) claim. Research conducted in high income countries has shown that indicators of disadvantage (or poverty) including socio-economic conditions, social disorganization, and other socio-structural factors reduce social cohesion, resulting in a breakdown of social networks and greater risk of sexual violence (Li et al., 2010).

Although measures of disadvantage consistently emerge as one of the determinants of sexual violence in nearly every study where they are assessed (Browning, 2002; Cunradi et al., 2000; Fox and Benson, 2006; Hindin and Adair, 2002; Khalifeh et al., 2013; Spriggs et al., 2009; Tyler and Johnson, 2006), key issues still remain regarding their measurement especially in research on low-income countries. Elaborating on this argument, Burns and Snow (2012) and Greif (2012) noted that the commonly used measures of disadvantage in existing research do not adequately reflect the realities in poor urban communities in sub-Saharan Africa. Moreover, the association between material deprivation and sexual risk is most frequently examined through the inclusion of either one dimension of deprivation (Burns and Snow, 2012) or a single deprivation-related item (Greif, 2012). It is likely that additional nuance underlying this association is not yet examined given that single-item measures of deprivations often overestimate or underestimate the statistical significance leading to biased results (Noble et al., 2010). In this paper, we use multiple-item measures of material deprivation – encompassing a lack of appropriate housing, food and healthcare – to assess relations among deprivation, SES and coercive sex.

### 2.2 A theoretical framework

Most of the existing research on relations between neighborhood characteristics and coercive sex is supported by theoretical perspectives positing that the health related behaviors of individuals are shaped not only by individual risk factors but also by attributes of places where people live (Brewster et al., 1993; Cohen et al., 2000; Cummins et al., 2007; Diez-Roux and Mair, 2010; Kawachi, 1999; Krieger and Higgins, 2002). Two approaches have been used to explain effects of place on health related behaviors. The first approach uses the idea that physical and social characteristics of a place shape health related behaviors of the whole group over and above the influence of aggregate attributes of individuals (Bernard et al., 2007; Cummins et al., 2007; Macintyre et al., 2002). The second approach, which is relevant for this study, uses the notion that geographical patterning of health outcomes is due to clustering of individuals with similar attributes (Bernard et al., 2007; Cummins et al., 2007; Macintyre et al., 2002). Accordingly, the association between place and health related behaviors is related to shared attributes among residents of a place (Bernard et al., 2007).

### 2.3 Study aim and hypothesis

Based on this guiding theoretical framework and our review of the literature we extend existing research by elaborating and testing hypotheses regarding the relationship between material deprivation, SES variables and coercive sex among both young men and young women residing in urban slums. Specifically, we explore whether a household-level material deprivation index affects coercive sex among young people in the two urban slums close to the two commercial centers, Blantyre and Limbe in Malawi. Accordingly, we hypothesize that the odds of coercive sex are higher amongst young people residing in materially-deprived households than non-deprived households.

## 3 Methods

### 3.1 Data

Data for this study come from a cross-sectional household survey conducted between June and July 2013 in Blantyre, Malawi. Participants were recruited through a three-stage sampling process in two purposively selected slum locations, Mbayani and Mtopwa, in Blantyre. In the first stage, 11 clusters, defined by geographic location, were selected. Households within selected clusters were systematically selected from a listed sampling frame. Every second or third household, depending on the cluster size, was selected for the survey. Eligible respondents identified in each selected household were 18–23 years old who spent the previous night in the household. One individual per household was selected using a Kish grid (Cramm et al., 2012) to ensure that all age-eligible individuals in the household stood an equal chance of being interviewed. Being a household survey, we excluded the homeless young people. Same-sex trained interviewers administered the pre-tested questionnaires in the relevant local language, Chichewa, in a private setting. The survey questionnaire collected a range of data including: demographics, household living conditions, socio-economic characteristics and sexual history. A sample size of 1123, adjusted for non-response or non-contact, was drawn to detect a between-group difference of 5% in sexual risk, with statistical significance of about 5% (*α*=0.05, two-tailed) and a power of more than 80% (1−*β*=0.80). The 5% between-group difference was chosen based on previous research (Mkandawire et al., 2011). We excluded 52 respondents from this sample due to inconsistencies in data reporting. Thus, a final sample of 1071 was used in this paper, representing a survey response rate of 92.1%. Ethics approval for the study was obtained from the University of the Witwatersrand Medical Research Ethics Committee and National Health Sciences Research Committee in Malawi (protocol numbers M120658 and 1078).

### 3.2 Description of variables

The outcome variable, coercive sex, was based on a binary response (1=coercive sex; 0=no coercive sex) from the item “Has anyone ever coerced or persuaded you to have sex when you did not want to?”

Material deprivation comprised three components (healthcare access, food and housing). The first component – poor access to healthcare – was measured with one item that asked young people, “Do you currently receive all the health care you think you need, or not?”

The second component, food deprivation, was measured by asking young people how often, in the last month, they worried that their household would not have enough food, how often they were not able to eat preferred foods because of a lack of resources, and whether anyone in the household went to sleep hungry. We used the responses to classify households into three groups: (1) severe food insecurity if at least one household member goes to sleep hungry or often worrying about food access or food quality; (2) some food insecurity if household members sometimes worried about food access or quality; and (3) food secure if household members experienced none of the conditions of (1) or (2).

Housing deprivation, the third component, was assessed with housing instability, quality and overcrowding. Housing instability was assessed using the following item: whether the respondent had stayed at or lived in an abandoned building for two or more days in last twelve months. Housing quality was captured as a dichotomous variable (0=temporary, 1=permanent), based on the wall, floor and roofing materials of the house; and the number of sleeping rooms. We measured overcrowding in terms of sleeping room occupancy ratio (number of people per sleeping room). Respondents in households with at least three people per sleeping room were considered as living in overcrowded houses.

We created a composite measure of material deprivation based on the three components that ranged from zero to three. Material deprivation was categorized as present if the sum was equal to zero or one and not present if the sum was equal to two or three.

We used three SES measures: household expenditure last month, household asset-based wealth, and a respondent׳s employment. Household monthly expenditures in Malawian Kwachas (MWK) were computed from the respondent׳s expenditures in the previous month. A household asset-based wealth index was created using a principal component analysis with a polychoric correlation matrix based on ownership of: radio, TV, cellular phone, bicycle, tables and chairs. We used the asset index to classify the households into tertiles – low, average and high. Employment was assessed by asking respondents their primary occupation – the work from which they earn most of their income. We created three categories from the responses: unemployed (0), self-employed (agriculture/services/trading) (1), and employed (construction/industry) (2).

Control variables^1^ included age in years, school status (in-school and out-of-school), highest level of education attained in years (range 0–17), duration of current residence (moved in past year, past 2–4 years, and always lived here), household structure (family with children, single with children, single/couple no children, and extended family), marital status (ever and never married), whether or not the respondent received money from their relatives and other health risk behaviors – young age at first sex, condom-use at last sex, multiple partners, age-difference with sexual partner, transactional sex and substance abuse as binary variables.

### 3.3 Statistical analysis

All analyses were conducted using STATA version 12. First, we present descriptive statistics of background and socio-economic characteristics of participants (Table 1), and material deprivations and risk behaviors of participants (Table 2). We used chi-square or *t*-tests for a comparison of place–place differentials (Table 3). We used bivariate logistic regression models to assess relationships between the outcome variable and each independent variable by place (Table 4). In Model 1 (Table 5), adjustments are made for the background characteristics and other risk behaviors and in Model 2 additionally for all the three SES measures. We checked for multi-collinearity among all independent variables included in Model 2 by examining the variance inflation factor, which were below the recommended cut-off of 10 (Agresti and Finlay, 2009). In Model 2, age was log-transformed to reduce kurtosis. Highest level of education was non-normal and transformed using the square root function.

### 3.4 Modelling framework

We apply the logit function that models the probability, Pr(CS_i_=1), of reporting coercive sex for an individual living in a household *i* nested within a neighborhood j approximated by the sampling cluster. It allows us to account for the hierarchical structure of the data. We fitted a two level random intercept model, with the individual as the first level and the neighborhood as the second level. The two-level random intercept model for individual living in household *i* nested within a neighborhood j may be represented as follows:

$$\mathrm{Pr}\left({\mathrm{CS}}_{i}=1\right)=\;\log\;{\mathrm{it}}^{-1}\left(ecstat_{j}\beta_{ecstat{\_}_{j}}+x_{i}^{I}\beta_{I}+\alpha_{k\left[i\right]}^{Neigh}\right)\alpha_{k[i]}^{Neigh}\sim \;N(\beta_{ecstat\_n}ecstat\_n_{j}+\beta_{matdev\_n}matdev\_n_{j}+x_{j}^{I}\beta_{N},\delta_{Neigh}^{2})$$

The model explains that the probability, Pr(CSi=1), of reporting coercive sex for an individual i, depends on household economic status, $ecstat_{j}$, other individual-level characteristics, $x_{i}^{I}$ and a neighborhood effect, $\alpha_{k[i]}^{Neigh}$. The neighborhood effect depends on economic status of a typical household and material deprivation, other neighborhood variables and an unexplained part. The unexplained part is assumed to be normally distributed with a homoscedastic variance and independent of regressors (Subramanian, 2004). However, we were unable to directly test neighborhood effects in the model because we are limited by the data. We also note that the number of clusters (*n*=11) in this paper is limited to permit meaningful analysis of neighborhood-level contextual factors, and detect significant effects on coercive sex. Thus, we introduced the household-level variables at the second level in the model.

## 4 Results

### 4.1 Descriptive results

Table 1 summarizes the sample descriptive statistics. Of the 1071 young people, 596 (55.6%) were male and 470 (43.9%) were in-school. Almost half (48.8%) of young men were out-of-school, compared with 65.3% of young women who were out of school. Participants were roughly equally distributed among the different categories for wealth and income, except for high numbers of unemployed young women (48.6%) and young men (26.3%).

Table 2 shows the prevalence of material deprivations and the sexual history of participants. Thirty-five percent of young men and 36.6% of young women reported some material deprivation. Nearly half (44.4%) of young women who ever had sex reported coercive sex, compared with 15.8% of young men.

### 4.2 Place–place differences of characteristics of the study participants

A summary of the behavioral, socio-demographic, and material deprivation characteristics of the study population is presented in Table 3. The focus here is on differences between the experiences of young people residing in different places in the same city; the urban slum (Mbayani) near Blantyre commercial centre and the urban slum (Mtopwa) close to Limbe commercial centre, highlighting examples of intra-urban inequalities and the role of place on these inequalities. Notably, young people residing in the urban slum near Blantyre commercial centre were significantly more likely to report coercive sex (38.9%; *n*=239 compared to 31.1%; *n*=142 of young people in the urban slum near Limbe; *χ*^2^=7.0; *p*<0.01). The other factors such as age of respondent, duration of residence and household composition were very similar between young people residing in Mbayani and Mtopwa. Young people residing in Mbayani were most likely to report living in very poor households (45.8%; *n*=281 compared to 24.0%; *n*=110 of young people in Mtopwa; *χ*^2^=100.6; *p*<0.0001). Young people residing in Mbayani were significantly more likely to report unemployment (42.4%; *n*=260 compared to 28.1%; *n*=128 of young people in the urban slum near Limbe; *χ*^2^=23.3; *p*<0.0001). However, a high proportion of young people in Mtopwa reported insufficient housing (82.3%; *n*=376), food insecurity (47.7%; *n*=218), and poor access to healthcare (39%; *n*=178).

### 4.3 Bivariate analysis of coercive sex and selected independent variables by place

Table 4 summarizes the result of the bivariate analyses of coercive sex and selected independent variables by place. Here we wish to highlight the role of place context in predicting experiences of coercive sex. With the exception of number of sexual partners, and age difference between partners – which were significantly related to coercive sex only in Mbayani – all risk behaviors were significantly related to coercive sex among young people residing in both Mbayani and Mtopwa. For young people in Mbayani, an older age of respondent was associated with lower odds of coercive sex (unadjusted OR=0.82, 95% CI: 0.76–0.89). Living in very poor households was significantly associated with likelihood of coercive sex in Mbayani (unadjusted OR=1.57, 95% CI: 1.14–2.17), but not in Mtopwa (unadjusted OR=1.31, 95% CI: 0.84–2.02). Unemployment was significantly associated with higher odds of reporting coercive sex in Mbayani (unadjusted OR=1.69, 95% CI: 1.30–2.18) and Mtopwa (unadjusted OR=1.90, 95% CI: 1.35–2.67). With respect to measures of material deprivation, poor access to healthcare was significantly associated with likelihood of coercive sex in Mbayani (unadjusted OR=1.74, 95% CI: 1.16–2.60), but not in Mtopwa (unadjusted OR=0.76, 95% CI: 0.55–1.06). This result is as expected considering that there was no health facility in Mbayani at the time of the study, while Mtopwa has a health facility nearby. There was a significant association between coercive sex and an aggregate measure of material deprivation in both places. In general, Table 4 indicates little difference between the two urban slums in terms of the association between measures of material deprivation and coercive sex, and the direction of association appears to be similar in the two urban slums.

### 4.4 Association of material deprivation and covariates with coercive sex

A look at Table 4 indicates little difference between the two urban slums in terms of the association between measures of material deprivation and coercive sex, and the direction of association appears to be similar. Thus, we considered the combination of data from the two urban slums and a material deprivation index as a variable in the multilevel model to examine the association between the place context and coercive sex. Results of the association of material deprivation and covariates with coercive sex are presented in Table 5 by gender. Among young men, when other risk behaviors, age, marital status, school status, and received money from relatives are controlled for (Model 1), material-deprivation was not significantly associated with coercive sex (OR=1.59, 95% CI: 0.90–2.82). The association between material deprivation and coercive sex remained insignificant when the three SES measures were added (Model 2) (OR=1.34, 95% CI: 0.75–2.39). However, young men who were unemployed had 77% (OR=1.77, 95% CI: 1.09–3.21) higher odds of reporting coercive sex than those who were working.

Among young women, Model 1 shows that young women who lived in materially-deprived households – net of other risk behaviors, age, marital status, duration of residence, household structure, school status, level of education and received money from relatives – were almost twice as likely to report coercive sex compared with those in non-materially deprived households (OR=1.40, 95% CI: 1.06–2.24). The association between material deprivation and coercive sex remained significant (OR=1.37, 95% CI: 1.07–2.22) when the three SES measures were added to the model (Model 2).

## 5 Discussion

The key findings of this study are as follows. Firstly, significant differences exist in the prevalence of coercive sex between the two urban slums in Blantyre, Malawi. Secondly, estimates from multi-level models suggest positive association between unemployment and coercive sex among young men. However, we found no association between material deprivation and coercive sex for young men in the adjusted multi-level models. This result suggests that facing hardship in meeting basic necessities does not seem to affect coercive sex for young men in urban slums. Lastly, this study has provided multi-level evidence of significant association between material deprivation and coercive sex among young women residing in two urban slums in Blantyre, Malawi. Put simply, this study has shown that some young women may be coerced to have sex for things deemed necessary for survival (such as housing, food or access to healthcare) – a notion that Tyler and Johnson (2006) described as coercive transactional sex in a qualitative study among homeless youth in the United States. However, we found no association between unemployment and coercive sex for young women in the adjusted multi-level models. Thus, our hypothesis holds true and our results support the claim by McIlwaine (2013) for young women.

The significant gender differences on the factors (material deprivation and unemployment) associated with coercive sex give insights to the different contexts within which coercive sex occurs for young women and young men in urban slums in Malawi. Additionally, these differences support the findings from other studies that the risk for coercive sex may differ between young men and young women, spurring researchers to improve measures of coercive sex among young men (Conroy and Chilungo, 2014; Erulkar, 2004; Jewkes et al., 2002; Moore et al., 2012; Sikweyiya and Jewkes, 2009).

Our finding that unemployment is associated with coercive sex for young men confirms those reported elsewhere (Gillham et al., 1998; Puri et al., 2010). An explanative pathway, for which there is support in the research literature, is that unemployment leads to depression, which is a risk factor for sexual violence (Puri et al., 2010), although this relationship has not been consistent for both genders. For example, while van Wijk and de Bruijn (2012) found that unemployment increased the risk for women׳s experience of sexual violence (but not for men), Jewkes et al. (2002) found no significant association between employment and sexual violence among women in South Africa. Many possible explanations can be given for these mixed research findings including lack of consistency in the measurement and definition of unemployment.

Our results have underscored the importance of utilizing the measures of deprivation of material resources, rather than SES indicators, in order to understand coercive sex among young women residing in urban slums. These results are similar to those found in research undertaken in high income countries where both measures of material deprivation and SES were considered in relation to partner sexual violence (Browning, 2002; Cunradi et al., 2000; Fox and Benson, 2006; Hindin and Adair, 2002; Khalifeh et al., 2013; Kiss et al., 2012; Spriggs et al., 2009). To explain why material deprivation, rather than measures of SES, is more important for explaining young women׳s coercive sex, it should be noted that deprivation of material resources is found to be a more proximate indicator of risk among women than income, education, and wealth (Cramm et al., 2012; Greif, 2012; Thomas et al., 2011; Vearey et al., 2010). Indeed in Malawi, narratives from focus group discussions and in-depth interviews with young people residing in urban slums add strength to the argument that whilst measures of SES have a positive influence on risk, it is essential to consider deprivation of material resources (Kamndaya et al., 2015). Additionally, our finding that material deprivation might be an important factor for young women, but not for men, conveys the notion that disadvantaged households in urban slums can exacerbate underlying gender-based power and socio-economic disparities, with young women subject to intensive gender-based sexual violence (Misganaw and Worku, 2013).

The study findings should be interpreted in light of several limitations. Firstly, the study relied on self-reporting by participants, which is subject to recall and reporting bias. Secondly, our measure of coercive sex was limited to a single question which might not capture the range of abusive sexual acts, including unwanted sexual touching or harassment, that young people may experience and that may predispose them to risky behaviors. Lastly, our study is based on cross-sectional data, making it impossible to assess causal effects.

Nevertheless, this study has contributed to an area of inquiry that has not received much attention – that of local indicators of material deprivation as important determinants of coercive sex among young people. Thus, using data from young people living in urban slums in Blantyre, Malawi, this study contributes to the extant literature in two main ways. First, by identifying individual-level factors that influence coercive sex among young people which to date has received less scholarly attention; and second, broadening the scholarship in this area by moving beyond individual risk factors to include contextual factors. A key strength of this paper is the use of local indicators of social environment and a diverse sample of young people for multi-level analysis of relations among material deprivation, SES and coercive sex in urban slums in Malawi.

## 6 Conclusion

We learn from this study that the joint use of local measures capturing material deprivation and SES is potentially fruitful for partitioning the effects of material deprivation and unemployment on coercive sex among young women and young men respectively. These findings have potential implications for strategies required for reducing coercive sex and its consequences (for example, HIV infection and unplanned pregnancy) among young people residing in slum settings in Malawi. For example, notwithstanding the growing evidence that cash transfer programs can reduce the risks of HIV infection and pregnancy among young people (Cluver et al., 2014), approaches for dealing with a lack of appropriate food, shelter, and health care in the growing urban slums need to be in place. Furthermore, research should pay more attention to analyses of indicators of the local social environment in order to develop locally-appropriate indicators for strategic interventions that have lasting impact for young people living in deprived neighborhoods. Future studies, using these locally-appropriate indicators, could investigate inter- and intra-place differences in relations among material deprivation, SES measures and coercive sex among young people across different locations – such as slums, medium density residential and low density high class residential locations in Malawi. Such a comparative study would increase our understanding of inter- and intra-place differentials in the associations between deprivation and coercive sex.

## Figures and Tables

**Table 1:** Distribution of background and socio-economic characteristics of the study participants.

**Characteristics**	**Young men**	**Young women**	**Total**
	*N* (%)	*N* (%)	*N* (%)
**Background characteristics**			
School status			
In-school	305 (51.2)	165 (34.7)	470 (43.9)
Out-of-school	291 (48.8)	310 (65.3)	601 (56.1)
Age (years)			
18–19	298 (50.0)	228 (48.0)	526 (49.1)
20–23	298 (50.0)	247 (52.0)	545 (50.9)
Marital status			
Never married	515 (86.4)	242 (51.0)	757 (70.7)
Ever married	81 (13.6)	233 (49.0)	314 (29.3)
Duration of residence			
Moved in past year	64 (10.7)	76 (16.0)	140 (13.1)
Moved in 2–4 years	131 (22.0)	91 (19.2)	222 (20.7)
Always lived here	401 (67.3)	308 (64.8)	709 (66.2)

**Socio-economic characteristics**			
Household structure			
Family with children	172 (28.9)	202 (42.5)	374 (34.9)
Single with children	126 (21.1)	77 (16.2)	203 (19.0)
Single/couple no children	63 (10.6)	37 (7.8)	100 (9.3)
Extended family	235 (39.4)	159 (33.5)	394 (36.8)
**Highest level of education** mean (SD)^a^	6.7±1.50	6.3±1.68	6.5±1.60
Received money from relatives last month (yes)	335 (56.2)	188 (39.6)	523 (48.8)
Household expenditure last month in 1000 MWK^b^ median (IQR)^c^	25 (IQR=25)	20 (IQR=25)	23 (IQR=22)
Wealth tertile			
Low	145 (23.3)	170 (35.8)	315 (29.4)
Average	186 (31.2)	179 (37.7)	365 (34.1)
High	265 (44.5)	126 (26.5)	391 (36.5)
Primary occupation			
Unemployed	157 (26.3)	231 (48.6)	388 (36.2)
Self-employed	380 (63.8)	237 (49.9)	617 (57.6)
Employed	59 (9.9)	7 (1.5)	66 (6.2)

a SD=standard deviation.

b MWK=Malawian Kwacha.

c IQR=Interquartile range.

**Table 2:** Prevalence of material deprivations and risk behaviors of the study participants.

**Variables**	**Young men**	**Young women**	**Total**
	*N* (%)	*N* (%)	*N* (%)
**Material deprivations**			
Housing quality			
Very low	145 (24.3)	170 (35.8)	315 (29.4)
Intermediate	186 (31.2)	179 (37.7)	365 (34.1)
High	265 (44.5)	126 (26.5)	391 (36.5)
Housing instability (yes)	124 (20.8)	63 (13.3)	187 (17.5)
**Household size** mean (SD)^a^	4.6±2.37	4.7±2.01	4.7±2.24
**Number of sleeping rooms** mean (SD)	2.3±1.03	2.1±0.97	2.2±1.02
**Number of rooms** mean (SD)	3.7±1.79	3.5±1.75	3.6±1.78
Overcrowding (yes)	248 (41.6)	266 (56.0)	514 (48.0)
Housing deprivation (yes)	388 (65.1)	342 (72.0)	730 (68.2)
Food insecurity (FI)			
Severe FI	183 (30.7)	148 (31.2)	331 (30.9)
Some FI	116 (19.5)	68 (14.3)	184 (17.2)
Food secure	297 (49.8)	259 (54.5)	556 (51.9)
Poor access to healthcare (yes)	168 (28.2)	112 (23.6)	280 (26.1)
Material deprivation (yes)	210 (35.2)	174 (36.6)	384 (35.9)

**Substance abuse and sexual history**			
Substance abuse (yes)	220 (36.9)	37 (7.8)	257 (24.0)
Ever had sex (yes)	544 (91.3)	417 (87.8)	961 (89.7)

**Among those who ever had sex**			
Age at first sex	14.4±1.88	14.4±2.00	14.4±1.93
Transactional sex (yes)	361 (66.4)	217 (52.0)	578 (60.2)
Partner ≥5 years older	69 (12.7)	126 (30.2)	195 (20.3)
2 or more sexual partners	268 (49.3)	78 (18.7)	346 (36.0)
No condom use at last sex	248 (45.6)	274 (65.7)	522 (54.3)
Coercive sex	86 (15.8)	185 (44.4)	271 (28.2)

a SD: standard deviation.

**Table 3:** A comparison of behavioral characteristics, socio-demographics, and material deprivations of study participants between the two urban slums.

**Variables**	**Mbayani**	**Mtopwa**	***χ***^**2**^**or*****t*****-test**	***p*****-Value**^a^
	***N*****(%)**	***N*****(%)**		
**Coercive sex** (yes)	239 (38.9)	142 (31.1)	7.0	<0.01
**Transactional sex in past 12 months**	314 (51.1)	264 (57.8)	4.6	<0.05
**2 or more sexual partners in past 12 months**	186 (30.3)	196 (42.9)	18.1	<0.0001
**Partner ≥5 years older**	93 (15.2)	102 (22.3)	9.1	<0.01
**No condom use at last sex**	336 (54.7)	296 (64.8)	10.9	<0.01
**Age at first sex** mean (SD)^b^	15.7±2.17	13.8±2.03	14.82	<0.0001
**Substance abuse**	172 (28.0)	85 (18.6)	12.7	<0.0001
**Age of respondent** mean (SD)	19.8±1.73	20.5±2.02	−6.40	<0.0001
**Female respondents**	248 (40.4)	227 (50.0)	9.1	<0.01
**Never married respondents**	540 (88.0)	217 (47.5)	207.0	<0.0001
**Duration of residence**			0.95	0.621
Always lived here	399 (65.0)	310 (68.0)		
1 year or less	83 (13.5)	57 (12.5)		
2–4 years	132 (21.5)	90 (19.7)		
**Household composition**			12.9	<0.05
Family, with children	209 (34.0)	165 (36.0)		
Single, with children	113 (18.4)	90 (19.7)		
Single/couple, no children	44 (7.2)	56 (12.3)		
Extended family	248 (40.4)	146 (32.0)		
**Out-of-school respondents**	227 (37.0)	374 (81.8)	214.2	<0.0001
**No money from relatives last month**	246 (40.1)	302 (66.1)	71.0	<0.0001
**Highest level of education** mean (SD)	7.1±1.28	5.7±1.62	16.0	<0.0001
**Expenditure last month** mean (SD)	35.9±25.41	20.5±21.47	10.32	<0.0001
**Wealth tertiles**			100.6	<0.0001
High	110 (17.9)	205 (44.9)		
Average	223 (36.3)	142 (31.1)		
Low	281 (45.8)	110 (24.0)		
**Unemployed**	260 (42.4)	128 (28.1)	23.3	<0.0001

**Deprivation measures**				
Housing deprivation (yes)	336 (59.6)	376 (82.3)	63.2	<0.0001
Food deprivation (yes)	114 (18.6)	218 (47.7)	104.0	<0.0001
Poor access to healthcare (yes)	102 (16.6)	178 (39.0)	67.7	<0.0001
**Material deprivation present**	131 (21.3)	265 (58.0)	151.0	<0.0001

a *p*<0.05: significance of the Pearson׳s *χ*^2^ test or *t*-test of the association between each variable of interest and urban slum location.

b SD: standard deviation.

**Table 4:** A bivariate analysis of coercive sex and selected independent variables by place.

**Independent variables**	**Unadjusted OR (95% CI)**	
	**Mbayani**	**Mtopwa**
**Transactional sex in past 12 months**	4.17 (3.10–5.59)⁎⁎⁎	1.52 (1.09–2.11)⁎
**2 or more sexual partners in past 12 months**	1.56 (1.15–2.11)⁎⁎	1.15 (0.82–1.61)
**Partner ≥5 years older**	1.26 (0.86–1.84)⁎⁎	1.15 (0.76–1.73)
**No condom use at last sex**	3.40 (2.52–4.59)⁎⁎⁎	1.91 (1.30–2.82)⁎⁎
**Age at first sex**	1.57 (1.43–1.73)⁎⁎⁎	1.10 (1.00–1.21)†
**Substance abuse**	2.52 (1.77–3.59)⁎⁎⁎	2.15 (1.26–3.68)⁎⁎
**Age of respondent**	0.82 (0.76–0.89)⁎⁎⁎	0.99 (0.91–1.07)
**Being female**	2.52 (1.94–3.27)⁎⁎⁎	3.54 (2.42–5.16)⁎⁎⁎

**Marital status**		
Never married (REF)	1.00	1.00
Ever married	0.77 (0.50–1.18)	1.01 (0.73–1.41)

**Duration of residence**		
Always (REF)	1.00	1.00
1 year or less	0.78 (0.52–1.16)	0.94 (0.55–1.60)
2–4 years	0.83 (0.60–1.15)	1.35 (0.91–2.00)

**Household composition**		
Family, with children (REF)	1.00	1.00
Single, with children	0.85 (0.59–1.23)	0.71 (0.44–1.13)
Single/couple, no children	0.89 (0.53–1.51)	0.38 (0.19–0.77)⁎
Extended family	0.85 (0.63–1.14)	0.84 (0.57–1.24)
**Out-of-school**	1.72 (1.30–2.30)⁎⁎⁎	1.71 (1.16–2.50)⁎⁎
**No money from relatives last month**	0.90 (0.69–1.17)	0.80 (0.57–1.13)
**Highest level of education**	1.04 (0.94–1.16)	0.92 (0.82–1.03)
**Household expenditure last month**	1.01 (1.00–1.01)⁎⁎	1.00 (0.99–1.01)

**Wealth tertiles**		
High (REF)	1.00	1.00
Average	1.08 (0.79–1.48)	1.15 (0.71–1.84)
Low	1.57 (1.14–2.17)⁎⁎	1.31 (0.84–2.02)
**Unemployed**	1.69 (1.30–2.18)⁎⁎⁎	1.90 (1.35–2.67)⁎⁎⁎

**Deprivation measures**		
Housing deprivation	1.21 (0.93–1.56)	1.24 (0.82–1.87)
Food deprivation	1.17 (0.83–1.65)	1.18 (0.84–1.64)
Poor access to healthcare	1.74 (1.16–2.60)⁎⁎	0.76 (0.55–1.06)
Material deprivation present	1.51 (1.07–2.14)⁎	1.14 (1.01–1.60)⁎

REF=Reference group.

† *p*<0.10.

⁎ *p*<0.05.

⁎⁎ *p*<0.01.

⁎⁎⁎ *p*<0.001.

**Table 5:** Association in stratified models between material deprivation and coercive sex.

**Variables**	**OR (95% CI)**^a^			
	**Young men**		**Young women**	
	**Model 1**	**Model 2**	**Model 1**	**Model 2**
**Material deprivation**				
Not present (REF)	1.00	1.00	1.00	1.00
Present	1.59 (0.90–2.82)	1.34 (0.75–2.39)	1.40 (1.06–1.84)⁎	1.37 (1.07–2.22)⁎
Transactional sex	0.33 (0.19–0.57)⁎⁎⁎	0.37 (0.21–0.65)⁎⁎⁎	0.86 (0.54–1.35)	0.81 (0.52–1.28)
Partner ≥5 years older	1.29 (0.50–3.32)	1.41 (0.55–3.61)	1.05 (0.66–1.67)	1.12 (0.70–1.78)
2 or more sexual partners	1.23 (0.73–2.07)	1.41 (0.81–2.44)	1.40 (0.82–2.41)	1.38 (0.81–2.35)
No condom at last sex	4.12 (2.46–6.90)⁎⁎⁎	4.52 (2.62–7.79)⁎⁎⁎	3.11 (1.86–5.21)⁎⁎⁎	3.20 (1.91–5.35)⁎⁎⁎
Age at first sex	1.34 (1.18–1.51)⁎⁎⁎	1.38 (1.21–1.59)⁎⁎⁎	1.11 (0.99–1.96)†	1.10 (0.99–1.23)†
Substance abuse	0.70 (0.42–1.18)	0.73 (0.42–1.27)	0.93 (0.44–1.96)	0.91 (0.43–1.92)
Age of respondent	0.92 (0.79–1.07)	0.94 (0.80–1.11)	1.09 (0.96–1.24)	1.06 (0.94–1.21)

**Marital status**				
Never married (REF)	1.00	1.00	1.00	1.00
Ever married	1.60 (0.60–4.26)	1.69 (0.64–4.49)	3.11 (1.63–5.97)⁎⁎⁎	2.79 (1.45–5.35)⁎⁎⁎
**Out-of-school**	1.98 (1.06–3.71)⁎	1.93 (0.97–3.85)!	1.95 (1.03–3.69)⁎⁎	2.06 (1.08–1.94)⁎
**No money from relatives last month**	1.04 (0.58–1.88)	1.03 (0.57–1.85)	0.98 (0.61–1.57)	0.98 (0.60–1.58)
**Expenditure last month**		1.01 (0.99–1.02)		0.99 (0.99–1.00)

**Wealth tertile**				
High (REF)		1.00		1.00
Average		0.67 (0.33–1.36)		1.01 (0.61–1.68)
Low		0.59 (0.29–1.21)		1.61(0.87–2.97)
**Unemployed**		1.77 (1.09–3.21)⁎		0.81 (0.53–1.24)
**Variance component**	0.17	0.18	0.17	0.17
**Intra class correlation**	0.08	0.09	0.08	0.08
**Sample size at level 1**	596	596	475	475
**Number of clusters**	11	11	11	11

a Odds ratio with 95% confidence interval.

† *p*<0.10.

⁎ *p*<0.05.

⁎⁎ *p*<0.01.

⁎⁎⁎ *p*<0.001.

## Footnotes

1 We performed multiple imputations via chained equations using the *mi ice* command although the fraction of missing on each covariate was 8% or less.

## References

[bib1] Adudans M.K., Montandon M., Kwena Z., Bukusi E.A., Cohen C.R. (2011). Prevalence of forced sex and associated factors among women and men in Kisumu, Kenya. Afr. J. Reprod. Health.

[bib2] Agresti A., Finlay B. (2009). Statistical Methods for the Social Sciences.

[bib3] Andersson N., Paredes-Solis S., Milne D., Omer K., Marokoane N., Laetsang D., Cockcroft A. (2012). Prevalence and risk factors for forced or coerced sex among school-going youth: national cross-sectional studies in 10 southern African countries in 2003 and 2007. BMJ Open.

[bib6] Bazargan-Hejazi S., Medeiros S., Mohammadi R., Lin J., Dalal K. (2013). Patterns of intimate partner violence: a study of female victims in Malawi. J. Inj. Violence Res..

[bib7] Bernard P., Charafeddine R., Frohlich K.L., Daniel M., Kestens Y., Potvin L. (2007). Health inequalities and place: a theoretical conception of neighbourhood. Soc. Sci. Med..

[bib8] Bingenheimer J.B., Reed E. (2014). Risk for coerced sex among female youth in Ghana: roles of family context, school enrollment and relationship experience. Int. Perspect. Sex. Reprod. Health.

[bib9] Brewster K.L., Billy J.O.G., Grady W.R. (1993). Social context and adolescent behavior: the impact of community on the transition to sexual activity. Soc. Forces.

[bib10] Browning C.R. (2002). The span of collective efficacy: extending social disorganization theory to partner violence. J. Marriage Fam..

[bib11] Burns P.A., Snow R.C. (2012). The built environment and the impact of neighborhood characteristics on youth sexual risk behavior in Cape Town, South Africa. Health Place.

[bib12] Chant S. (2013). Cities through a “gender lens”: a golden “urban age” for women in the global South?. Environ. Urban..

[bib13] Chipeta L. (2009). The water crisis in Blantyre City and its impact on women: the cases of Mbayani and Mtopwa, Malawi. J. Int. Women Stud..

[bib14] Cluver L.D., Orkin F.M., Boyes M.E., Sherr L. (2014). Cash plus care: social protection cumulatively mitigates HIV-risk behaviour among adolescents in South Africa. AIDS.

[bib15] Cohen D., Spear S., Scribner R., Kissinger P., Mason K., Wildgen J. (2000). “Broken windows” and the risk of gonorrhea. Am. J. Public Health.

[bib16] Conroy A.A., Chilungo A. (2014). Male victims of sexual violence in rural Malawi: the overlooked association with HIV infection. AIDS Care.

[bib17] Cramm J., Møller V., Nieboer A. (2012). Individual- and neighborhood-level indicators of subjective well-being in a small and poor Eastern Cape township: the effect of health, social capital, marital status, and income. Soc. Indic. Res..

[bib18] Cummins S., Curtis S., Diez-Roux A.V., Macintyre S. (2007). Understanding and representing ‘place’ in health research: a relational approach. Soc. Sci. Med..

[bib19] Cunradi C.B., Caetano R., Clark C., Schafer J. (2000). Neighborhood poverty as a predictor of intimate partner violence among white, black, and hispanic couples in the United States: a multilevel analysis. Ann. Epidemiol..

[bib20] Decker M.R., Peitzmeier S., Olumide A., Acharya R., Ojengbede O., Covarrubias L., Gao E., Cheng Y., Delany-Moretlwe S., Brahmbhatt H. (2014). Prevalence and health impact of intimate partner violence and non-partner sexual violence among female adolescents aged 15–19 years in vulnerable urban environments: a multi-country study. J. Adolesc. Health.

[bib21] Diez-Roux A.V., Mair C. (2010). Neighborhoods Health. ..

[bib22] Erulkar A.S. (2004). The experience of sexual coercion among young people in Kenya. Int. Fam. Plan. Perspect..

[bib23] Fox G.L., Benson M.L. (2006). Household and neighborhood contexts of intimate partner violence. Public Health Rep..

[bib24] Frye V., Blaney S., Cerdá M., Vlahov D., Galea S., Ompad D.C. (2014). Neighborhood characteristics and sexual intimate partner violence against women among low-income, drug-involved New York City residents: results from the IMPACT studies. Violence Against Women.

[bib25] Gillham B., Tanner G., Cheyne B., Freeman I., Rooney M., Lambie A. (1998). Unemployment rates, single parent density, and indices of child poverty: their relationship to different categories of child abuse and neglect. Child Abuse Negl..

[bib26] Greif M.J. (2012). Housing, medical and food deprivation in poor urban contexts: implications for multiple sexual partnerships and transactional sex in Nairobi׳s slums. Health Place.

[bib27] Hindin M.J., Adair L.S. (2002). Who׳s at risk? Factors associated with intimate partner violence in the Philippines. Soc. Sci. Med..

[bib28] Hines D. (2007). Predictors of sexual coercion against women and men: a multilevel, multinational study of university students. Arch. Sex Behav..

[bib29] Jewkes R. (2002). Intimate partner violence: causes and prevention. Lancet.

[bib30] Jewkes R., Levin J., Penn-Kekana L. (2002). Risk factors for domestic violence: findings from a South African cross-sectional study. Soc. Sci. Med..

[bib4] Kamndaya M., Thomas L., Vearey J., Sartorius B., Kazembe L. (2014). Material deprivation affects high sexual risk behavior among young people in urban slums, South Africa. J. Urban Health.

[bib5] Kamndaya M., Vearey J., Thomas L., Kabiru C.W., Kazembe L.N. (2015). The role of material deprivation and consumerism in the decisions to engage in transactional sex among young people in the urban slums of Blantyre, Malawi. Glob. Public Health.

[bib31] Kabiru C.W., Beguy D., Crichton J., Ezeh A. (2010). Self-reported drunkenness among adolescents in four sub-Saharan African countries: associations with adverse childhood experiences. Child Adolesc. Psychiatry Ment. Health.

[bib32] Kathewera-Banda M., Gomile-Chidyaonga F., Hendriks S., Kachika T., Mitole Z., White S. (2005). Sexual violence and women׳s vulnerability to HIV transmission in Malawi: a rights issue. Int. Soc. Sci. J..

[bib33] Kawachi I. (1999). Social capital and community effects on population and individual health. Ann. N. Y. Acad. Sci..

[bib34] Khalifeh H., Hargreaves J., Howard L.M., Birdthistle I. (2013). Intimate partner violence and socioeconomic deprivation in England: findings from a national cross-sectional survey. Am. J. Public Health.

[bib35] Kiss L., Schraiber L.B., Heise L., Zimmerman C., Gouveia N., Watts C. (2012). Gender-based violence and socioeconomic inequalities: does living in more deprived neighbourhoods increase women׳s risk of intimate partner violence?. Soc. Sci. Med..

[bib36] Koenig M.A., Lutalo T., Zhao F., Nalugoda F., Kiwanuka N., Wabwire-Mangen F., Kigozi G., Sewankambo N., Wagman J., Serwadda D., Wawer M., Gray R. (2004). Coercive sex in rural Uganda: prevalence and associated risk factors. Soc. Sci. Med..

[bib37] Krieger J., Higgins D.L. (2002). Housing and health: time again for public health action. Am. J. Public Health.

[bib38] Kuyper L., de Wit J., Smolenski D., Adam P., Woertman L., van Berlo W. (2013). Gender differences in patterns of experienced sexual coercion and associated vulnerability factors among young people in the Netherlands. J. Interpers. Violence.

[bib39] Li Q., Kirby R.S., Sigler R.T., Hwang S.-S., LaGory M.E., Goldenberg R.L. (2010). A multilevel analysis of individual, household, and neighborhood correlates of intimate partner violence among low-income pregnant women in Jefferson County, Alabama. Am. J. Public Health.

[bib40] Macintyre S., Ellaway A., Cummins S. (2002). Place effects on health: how can we conceptualise, operationalise and measure them?. Soc. Sci. Med..

[bib41] Mandalazi P., Banda C., Umar E. (2013). Street children׳s vulnerability to HIV and sexually transmitted infections in Malawian cities. Malawi Med. J..

[bib42] Mason C., Kennedy N. (2014). Sexual abuse in Malawi: patterns of disclosure. J. Child Sex. Abuse.

[bib43] McCrann D., Lalor K., Katabaro J.K. (2006). Childhood sexual abuse among university students in Tanzania. Child Abuse Negl..

[bib44] McIlwaine C. (2013). Urbanization and gender-based violence: exploring the paradoxes in the global South. Environ. Urban..

[bib45] Meinck F., Cluver L.D., Boyes M.E., Mhlongo E.L. (2015). Risk and protective factors for physical and sexual abuse of children and adolescents in Africa: a review and implications for practice. Trauma Violence Abuse.

[bib46] Misganaw A.C., Worku Y.A. (2013). Assessment of sexual violence among street females in Bahir-Dar town, North West Ethiopia: a mixed method study. BMC Public Health.

[bib47] Mkandawire P., Luginaah I., Tobias J. (2011). Landscapes of economic deprivation and locally distilled liquor (kachasu): an emerging milieu of HIV/AIDS risk in urban Northern Malawi. Environ. Plan..

[bib48] Moore A.M., Awusabo-Asare K., Madise N., John-Langba J., Kumi-Kyereme A. (2007). Coerced first sex among adolescent girls in sub-Saharan Africa: prevalence and context. Afr. J. Reprod. Health.

[bib49] Moore A.M., Madise N., Awusabo-Asare K. (2012). Unwanted sexual experiences among young men in four sub-Saharan African countries: prevalence and context. Cult. Health Sex..

[bib50] Mughogho B., Kosamu L. (2012). Water supply arrangements in developing countries: a case study of Blantyre City, Malawi. Afr. J. Environ. Sci. Technol..

[bib51] Mulumeoderhwa M., Harris G. (2014). Forced sex, rape and sexual exploitation: attitudes and experiences of high school students in South Kivu, Democratic Republic of Congo. Cult. Health Sex..

[bib52] National Statistical Office of Malawi. 2008. 2008 Population and Housing Census Results [Online]. National Statistical Office of Malawi, Zomba. Available from: 〈http://www.nsomalawi.mw/〉 (accessed 30.12.13).

[bib53] National Statistical Office of Malawi and ICF Macro (2011). Malawi Demographic and Health Survey 2010.

[bib54] Noble M., Barnes H., Wright G., Roberts B. (2010). Small area indices of multiple deprivation in South Africa. Soc. Indic. Res..

[bib55] Oduro G.Y., Swartz S., Arnot M. (2012). Gender-based violence: young women׳s experiences in the slums and streets of three sub-Saharan African cities. Theory Res. Educ..

[bib56] Pandey G.K., Dutt D., Banerjee B. (2009). Partner and relationship factors in domestic violence: perspectives of women from a slum in Calcutta, India. J. Interpers. Violence.

[bib57] Pearlman D.N., Zierler S., Gjelsvik A., Verhoek-Oftedahl W. (2003). Neighborhood environment, racial position, and risk of police-reported domestic violence: a contextual analysis. Public Health Rep..

[bib58] Pinchevsky G.M., Wright E.M. (2012). The impact of neighborhoods on intimate partner violence and victimization. Trauma Violence Abuse.

[bib59] Population Reference Bureau [PRB] (2013). World Population Data Sheet.

[bib60] Puri M., Shah I., Tamang J. (2010). Exploring the nature and reasons for sexual violence within marriage among young women in Nepal. J. Interpers. Violence.

[bib61] Sikweyiya Y., Jewkes R. (2009). Force and temptation: contrasting South African men׳s accounts of coercion into sex by men and women. Cult. Health Sex..

[bib62] Spriggs A.L., Halpern C.T., Herring A.H., Schoenbach V.J. (2009). Family and school socioeconomic disadvantage: Interactive influences on adolescent dating violence victimization. Soc. Sci. Med..

[bib63] Subramanian S.V. (2004). The relevance of multilevel statistical methods for identifying causal neighborhood effects. Soc. Sci. Med..

[bib64] Thomas L., Vearey J., Mahlangu P. (2011). Making a difference to health in slums: an HIV and African perspective. Lancet.

[bib65] Tyler K.A., Johnson K.A. (2006). Trading sex: voluntary or coerced? The experiences of homeless youth. J*.* Sex Res..

[bib66] UN-Habitat, 2011. Malawi: Blantyre Urban Profile. UN-Habitat [Online]. UN-Habitat, Blantyre. Available from: 〈http://mirror.unhabitat.org/pmss/listItemDetails.aspx?publicationID=3172〉 (accessed 11.01.14.).

[bib67] van Wijk N.P., de Bruijn J.G. (2012). Risk factors for domestic violence in Curacao. J. Interpers. Violence.

[bib68] Vearey J., Palmary I., Thomas L., Nunez L., Drimie S. (2010). Urban health in Johannesburg: the importance of place in understanding intra-urban inequalities in a context of migration and HIV. Health Place.

[bib69] World Bank, 2010. Malawi at a Glance [Online]. Available from: 〈http://devdata.worldbank.org/AAG/mwi_aag.pdf〉 (accessed 14.06.14.).

[bib70] World Health Organization (2013). Global and regional estimates of violence against women: prevalence and health effects of intimate partner violence and non-partner sexual violence.

[bib71] Ybarra M.L., Bull S.S., Kiwanuka J., Bangsberg D.R., Korchmaros J. (2012). Prevalence rates of sexual coercion victimization and perpetration among Uganda adolescents. AIDS Care.

[bib72] Zablotska I.B., Gray R.H., Koenig M.A., Serwadda D., Nalugoda F., Kigozi G., Sewankambo N., Lutalo T., Mangen F.W., Wawer M. (2009). Alcohol use, intimate partner violence, sexual coercion and HIV among women aged 15–24 in Rakai, Uganda. AIDS Behav..

